# (4*S*,8*S*,9*R*,12*E*)-8,9,16,18-Tetrahydroxy-4-methyl-3-oxabicyclo[12.4.0]octadeca-12,14,16,18-tetraen-2-one monohydrate

**DOI:** 10.1107/S1600536808010258

**Published:** 2008-05-07

**Authors:** Ling-Ling Zhao, Yue Gai, Hisayoshi Kobayashi, Chang-Qi Hu, Hui-Ping Zhang

**Affiliations:** a Department of Natural Products Chemistry, School of Pharmacy, Fudan University, Shanghai 200032, People’s Republic of China; b Institute of Molecular and Cellular Biosciences, The University of Tokyo, Tokyo 113-0032, Japan

## Abstract

The asymmetric unit of the title compound, C_18_H_24_O_6_·H_2_O, contains a 14-membered macrolide mol­ecule and a water mol­ecule. In the crystal structure, intra­molecular C—H⋯O and O—H⋯O hydrogen bonds help to stabilize the mol­ecular conformation, while inter­molecular O—H⋯O hydrogen bonds link the mol­ecules, forming an infinite network. The absolute configuration was assigned by comparison with related zearalenone compounds, but needs verification.

## Related literature

For the extraction of the components of *Fusarium* sp. 05ABR26 see: Zhao *et al.* (2008 ▶). For the crystal structure of zearalenol, see: Gelo-Pujić *et al.* (1994 ▶). For related zearalenone series compounds, see: Zinedine *et al.* (2007 ▶).  For bond-length data, see: Allen *et al.* (1987 ▶).
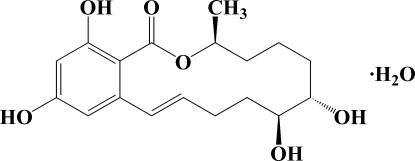


## Experimental

### 

#### Crystal data

C_18_H_24_O_6_·H_2_O
*M* *_r_* = 354.39
Monoclinic, 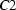

*a* = 18.23 (1) Å
*b* = 8.078 (6) Å
*c* = 13.86 (1) Å
β = 118.441 (7)°
*V* = 1795 (2) Å^3^
*Z* = 4
Mo *K*α radiation
μ = 0.10 mm^−1^
*T* = 293 (2) K
0.20 × 0.15 × 0.05 mm

#### Data collection

Bruker SMART APEX CCD area-detector diffractometer
Absorption correction: multi-scan (*SADABS*; Sheldrick, 1996 ▶) *T*_min_ = 0.980, *T*_max_ = 0.995
4458 measured reflections
2076 independent reflections
1804 reflections with *I* > 2σ(*I*)
*R*_int_ = 0.081

#### Refinement

*R*[*F* ^2^ > 2σ(*F* ^2^)] = 0.060
*wR*(*F* ^2^) = 0.145
*S* = 1.03
2076 reflections
239 parameters
4 restraints
H atoms treated by a mixture of independent and constrained refinement
Δρ_max_ = 0.28 e Å^−3^
Δρ_min_ = −0.36 e Å^−3^

### 

Data collection: *SMART* (Bruker, 2000 ▶); cell refinement: *SMART*; data reduction: *SAINT* (Bruker, 2000 ▶); program(s) used to solve structure: *SHELXS97* (Sheldrick, 2008 ▶); program(s) used to refine structure: *SHELXL97* (Sheldrick, 2008 ▶); molecular graphics: *SHELXTL* (Sheldrick, 2008 ▶); software used to prepare material for publication: *SHELXTL*.

## Supplementary Material

Crystal structure: contains datablocks global, I. DOI: 10.1107/S1600536808010258/im2058sup1.cif

Structure factors: contains datablocks I. DOI: 10.1107/S1600536808010258/im2058Isup2.hkl

Additional supplementary materials:  crystallographic information; 3D view; checkCIF report

## Figures and Tables

**Table 1:** Hydrogen-bond geometry (Å, °)

*D*—H⋯*A*	*D*—H	H⋯*A*	*D*⋯*A*	*D*—H⋯*A*
O1—H1⋯O3	0.82	1.85	2.560 (4)	144
O2—H2⋯O7	0.82	1.79	2.608 (4)	172
C3—H3⋯O7	0.93	2.77	3.398 (4)	125
C9—H9*B*⋯O3	0.96	2.73	3.198 (5)	111
O1—H1⋯O3^i^	0.82	2.46	3.003 (4)	125
O7—H7*Y*⋯O2^ii^	0.844 (18)	2.01 (3)	2.798 (4)	156 (6)
O5—H5⋯O6^iii^	0.82	2.02	2.821 (4)	166
O6—H6⋯O1^iv^	0.82	2.13	2.871 (4)	149
C3—H3⋯O6^v^	0.93	2.88	3.398 (4)	116
C10—H10*B*⋯O2^v^	0.97	2.52	3.346 (6)	144
O7—H7*X*⋯O5^vi^	0.857 (18)	1.818 (19)	2.674 (4)	176 (4)

Symmetry codes: (i) 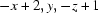; (ii) 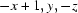; (iii) 
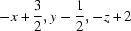
; (iv) 
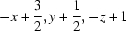
; (v) 
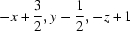
; (vi) 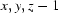.

**Fig. 1.**
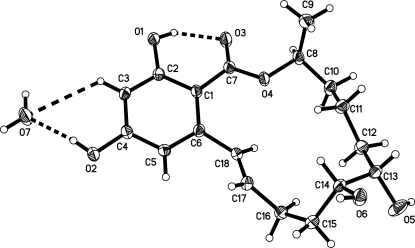
A view of the asymmetric unit of 5'-hydroxyzearalenol monohydrate showing the atom-labelling scheme. Displacement ellipsoids are drawn at the 30% probability level. Hydrogen bonds are drawn as dashed lines.

**Fig. 2.**
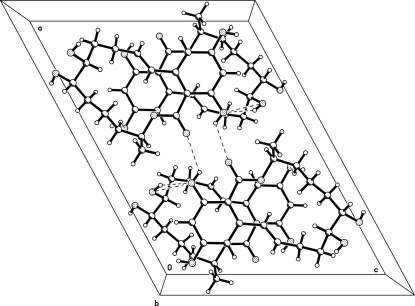
The molecular packing diagram of 5'-hydroxyzearalenol monohydrate viewed down the b axis. Hydrogen bonds are shown as dashed lines.

## supplementary crystallographic information

### Comment

The title compound, (I), 5'-hydroxyzearalenol is a new natural β-resorcylic macrolide which has recently been isolated (Zhao *et al.*, 2008) from the culture of a marine-derived fungus *Fusarium sp*. 05ABR26. It is closely related in structure to the zearalenone series compounds (Zinedine *et al.*, 2007), which show attractive cytotoxic and genotoxic effects. As a continuation of our studies on the secondary metabolites of *Fusarium sp*. 05ABR26, we report here the crystal structure of 5'-hydroxyzearalenol monohydrate.

In the asymmetric unit of (I) one molecule of 5'-hydroxyzearalenol and one solvent water molecule are observed. The β-resorcylic unit (C1—C7) is essentially planar, with a r.m.s. deviation of the respective atoms of 0.010 (4) Å. The 14-membered macrolide (C1/C6—C8/C10—C18/O4) adopts a twist conformation which is additionally stabilized by weak intramolecular C—H···O and O—H···O hydrogen bond (Table 1). Otherwise, all bond lengths and angles are within normal ranges (Allen *et al.*, 1987).

The structure of (I) is built up from the self-assembly of the molecules of 5'-hydroxyzearalenol with water molecules *via* hydrogen-bond interactions. The water molecule is involved as a donor and accepter of hydrogen bonds. The crystal structure is stabilized by intermolecular O—H···O hydrogen bonds (Fig. 2 and Table 1).

It was not possible to accurately determine the absolute configuration of (I) by anomalous dispersion effects in the case of using Mo K/a radiation (0.71073 Å). However, the naturally occurring compounds of the zearalenone series all had the same C3*S* configuration (Zinedine *et al.*, 2007), thereofore leading to the assignment of the absolute configurations of C7 and C8 to be *S* and *R*, respectively. Nevertheless, this absolute configuration of the molecule needs further verifcatio.

### Experimental

5'-hydroxyzearalenol was isolated from 1*L* culture of a marine-derived fungus 05ABR26 (a *Fusarium sp*.), affording 9.1 mg by repeated column chromatography on Sephadex LH-20 and Silica gel. Single crystals suitable for X-ray analysis were grown by slow evaporation of a solution of 5'- hydroxyzearalenol in n-hexane:acetone (3:1 *v*/*v*) at room temperature.

### Refinement

The H atoms of the water molecule were located in a different Fourier map. The remaining H atoms were positioned geometrically and allowed to ride on their parent atoms, with O—H distance of 0.82 Å and C—H distances in the range 0.93–0.98 Å. The *U*_iso_(H) values were constrained to be 1.5*U*_eq_(carrier atom) for hydroxy and methyl H atoms and 1.2*U*_eq_(carrier atom) for the remaining H atoms.

### Crystal data

**Table:**

C_18_H_24_O_6_·H_2_O	*F*(000) = 760
*M**_r_* = 354.39	*D*_x_ = 1.312 Mg m^−^^3^
Monoclinic, *C*2	Mo *K*α radiation, λ = 0.71073 Å
Hall symbol: C 2y	Cell parameters from 687 reflections
*a* = 18.23 (1) Å	θ = 2.3–26.4°
*b* = 8.078 (6) Å	µ = 0.10 mm^−^^1^
*c* = 13.86 (1) Å	*T* = 293 K
β = 118.441 (7)°	Prismatic, colorless
*V* = 1795 (2) Å^3^	0.20 × 0.15 × 0.05 mm
*Z* = 4	

### Data collection

**Table:**

Bruker SMART APEX CCD area-detector diffractometer	2076 independent reflections
Radiation source: fine-focus sealed tube	1804 reflections with *I* > 2σ(*I*)
graphite	*R*_int_ = 0.081
φ and ω scans	θ_max_ = 27.0°, θ_min_ = 1.7°
Absorption correction: multi-scan (*SADABS*; Sheldrick, 1996)	*h* = −23→22
*T*_min_ = 0.980, *T*_max_ = 0.995	*k* = −10→4
4458 measured reflections	*l* = −17→17

### Refinement

**Table:**

Refinement on *F*^2^	Primary atom site location: structure-invariant direct methods
Least-squares matrix: full	Secondary atom site location: difference Fourier map
*R*[*F*^2^ > 2σ(*F*^2^)] = 0.060	Hydrogen site location: inferred from neighbouring sites
*wR*(*F*^2^) = 0.145	H atoms treated by a mixture of independent and constrained refinement
*S* = 1.03	*w* = 1/[σ^2^(*F*_o_^2^) + (0.0914*P*)^2^] where *P* = (*F*_o_^2^ + 2*F*_c_^2^)/3
2076 reflections	(Δ/σ)_max_ < 0.001
239 parameters	Δρ_max_ = 0.28 e Å^−^^3^
4 restraints	Δρ_min_ = −0.36 e Å^−^^3^

### Special details

**Table:**

Geometry. All e.s.d.'s (except the e.s.d. in the dihedral angle between two l.s. planes) are estimated using the full covariance matrix. The cell e.s.d.'s are taken into account individually in the estimation of e.s.d.'s in distances, angles and torsion angles; correlations between e.s.d.'s in cell parameters are only used when they are defined by crystal symmetry. An approximate (isotropic) treatment of cell e.s.d.'s is used for estimating e.s.d.'s involving l.s. planes.
Refinement. Refinement of *F*^2^ against ALL reflections. The weighted *R*-factor *wR* and goodness of fit *S* are based on *F*^2^, conventional *R*-factors *R* are based on *F*, with *F* set to zero for negative *F*^2^. The threshold expression of *F*^2^ > σ(*F*^2^) is used only for calculating *R*-factors(gt) *etc*. and is not relevant to the choice of reflections for refinement. *R*-factors based on *F*^2^ are statistically about twice as large as those based on *F*, and *R*- factors based on ALL data will be even larger.

### Fractional atomic coordinates and isotropic or equivalent isotropic displacement parameters (Å^2^)

**Table:**

	*x*	*y*	*z*	*U*_iso_*/*U*_eq_	
O1	0.87040 (13)	0.9829 (4)	0.35095 (16)	0.0406 (6)	
H1	0.9091	0.9490	0.4085	0.061*	
O2	0.57709 (13)	1.0102 (4)	0.17058 (17)	0.0424 (6)	
H2	0.5857	1.0188	0.1179	0.064*	
O3	0.94597 (14)	0.9208 (5)	0.55690 (19)	0.0555 (9)	
O4	0.88077 (12)	0.9851 (4)	0.65316 (15)	0.0393 (6)	
O5	0.72723 (16)	1.0005 (5)	0.9735 (2)	0.0633 (10)	
H5	0.7609	0.9364	1.0189	0.095*	
O6	0.66693 (14)	1.3026 (3)	0.84561 (17)	0.0397 (6)	
H6	0.6404	1.3623	0.7923	0.060*	
C1	0.79948 (18)	0.9669 (4)	0.4619 (2)	0.0303 (7)	
C2	0.79927 (18)	0.9842 (4)	0.3594 (2)	0.0323 (6)	
C3	0.72615 (18)	1.0056 (4)	0.2630 (2)	0.0325 (7)	
H3	0.7275	1.0229	0.1975	0.039*	
C4	0.65057 (18)	1.0013 (4)	0.2639 (2)	0.0329 (7)	
C5	0.64859 (18)	0.9820 (5)	0.3630 (2)	0.0339 (7)	
H5A	0.5974	0.9807	0.3626	0.041*	
C6	0.72118 (18)	0.9649 (4)	0.4615 (2)	0.0307 (6)	
C7	0.88176 (18)	0.9540 (5)	0.5598 (2)	0.0348 (8)	
C8	0.95973 (17)	0.9717 (5)	0.7566 (2)	0.0371 (8)	
H8	0.9929	0.8813	0.7501	0.044*	
C9	1.0079 (2)	1.1292 (6)	0.7784 (3)	0.0518 (10)	
H9A	0.9748	1.2192	0.7817	0.078*	
H9B	1.0214	1.1488	0.7204	0.078*	
H9C	1.0583	1.1207	0.8470	0.078*	
C10	0.9324 (2)	0.9239 (5)	0.8403 (3)	0.0403 (8)	
H10A	0.9817	0.9075	0.9107	0.048*	
H10B	0.9031	0.8190	0.8186	0.048*	
C11	0.8761 (2)	1.0503 (5)	0.8547 (3)	0.0390 (8)	
H11A	0.8391	1.0999	0.7843	0.047*	
H11B	0.9100	1.1376	0.9037	0.047*	
C12	0.8244 (2)	0.9678 (5)	0.9017 (3)	0.0384 (8)	
H12A	0.7921	0.8786	0.8531	0.046*	
H12B	0.8621	0.9189	0.9721	0.046*	
C13	0.76515 (19)	1.0832 (5)	0.9174 (2)	0.0372 (8)	
H13	0.7985	1.1746	0.9643	0.045*	
C14	0.69633 (19)	1.1591 (4)	0.8122 (2)	0.0319 (7)	
H14	0.7218	1.1976	0.7680	0.038*	
C15	0.6218 (2)	1.0502 (5)	0.7392 (3)	0.0402 (8)	
H15A	0.6040	0.9934	0.7861	0.048*	
H15B	0.5766	1.1225	0.6914	0.048*	
C16	0.6333 (2)	0.9202 (5)	0.6675 (3)	0.0394 (8)	
H16A	0.6779	0.8464	0.7150	0.047*	
H16B	0.5826	0.8549	0.6320	0.047*	
C17	0.65289 (19)	0.9846 (5)	0.5803 (2)	0.0365 (7)	
H17	0.6211	1.0723	0.5372	0.044*	
C18	0.71206 (18)	0.9253 (4)	0.5608 (2)	0.0320 (7)	
H18	0.7509	0.8537	0.6124	0.038*	
O7	0.58905 (17)	1.0465 (9)	−0.0084 (2)	0.104 (2)	
H7X	0.6347 (15)	1.031 (7)	−0.011 (3)	0.065 (14)*	
H7Y	0.5433 (15)	1.057 (9)	−0.067 (3)	0.084 (17)*	

### Atomic displacement parameters (Å^2^)

**Table:**

	*U*^11^	*U*^22^	*U*^33^	*U*^12^	*U*^13^	*U*^23^
O1	0.0283 (10)	0.0658 (18)	0.0285 (10)	0.0009 (12)	0.0143 (9)	−0.0025 (13)
O2	0.0303 (10)	0.0656 (19)	0.0266 (10)	0.0022 (12)	0.0099 (9)	0.0024 (12)
O3	0.0308 (11)	0.105 (3)	0.0311 (11)	0.0088 (14)	0.0150 (10)	−0.0045 (14)
O4	0.0271 (9)	0.0652 (16)	0.0222 (10)	0.0050 (12)	0.0088 (8)	−0.0037 (12)
O5	0.0505 (15)	0.096 (3)	0.0577 (16)	0.0275 (17)	0.0372 (14)	0.0453 (18)
O6	0.0403 (13)	0.0473 (14)	0.0316 (11)	0.0104 (11)	0.0172 (11)	0.0001 (11)
C1	0.0296 (13)	0.0349 (18)	0.0239 (12)	0.0002 (13)	0.0108 (11)	−0.0028 (13)
C2	0.0337 (14)	0.0363 (17)	0.0280 (13)	−0.0036 (15)	0.0156 (12)	−0.0052 (15)
C3	0.0355 (15)	0.0392 (18)	0.0258 (12)	0.0005 (14)	0.0170 (12)	−0.0004 (13)
C4	0.0308 (14)	0.0371 (18)	0.0265 (13)	0.0024 (14)	0.0102 (12)	−0.0022 (13)
C5	0.0271 (13)	0.0457 (18)	0.0303 (14)	0.0007 (14)	0.0147 (12)	−0.0017 (15)
C6	0.0328 (14)	0.0345 (17)	0.0261 (13)	0.0015 (13)	0.0150 (12)	−0.0022 (12)
C7	0.0297 (14)	0.048 (2)	0.0275 (14)	0.0016 (14)	0.0139 (12)	−0.0025 (14)
C8	0.0240 (13)	0.057 (2)	0.0248 (13)	0.0109 (15)	0.0072 (12)	0.0000 (15)
C9	0.0408 (19)	0.071 (3)	0.047 (2)	−0.0069 (19)	0.0238 (18)	−0.013 (2)
C10	0.0356 (16)	0.053 (2)	0.0302 (15)	0.0129 (16)	0.0139 (13)	0.0081 (15)
C11	0.0377 (16)	0.045 (2)	0.0397 (16)	0.0036 (15)	0.0230 (15)	0.0020 (15)
C12	0.0326 (14)	0.045 (2)	0.0354 (15)	0.0080 (15)	0.0141 (13)	0.0100 (15)
C13	0.0317 (15)	0.054 (2)	0.0304 (15)	0.0018 (15)	0.0182 (14)	0.0059 (14)
C14	0.0310 (14)	0.0415 (18)	0.0287 (14)	0.0015 (13)	0.0187 (13)	0.0002 (13)
C15	0.0379 (16)	0.053 (2)	0.0370 (16)	−0.0036 (16)	0.0234 (15)	−0.0040 (16)
C16	0.0408 (17)	0.0452 (19)	0.0349 (16)	−0.0130 (16)	0.0202 (15)	−0.0087 (15)
C17	0.0375 (15)	0.0431 (18)	0.0295 (14)	−0.0029 (15)	0.0165 (13)	−0.0035 (15)
C18	0.0291 (14)	0.0382 (17)	0.0247 (14)	−0.0044 (13)	0.0096 (12)	−0.0011 (12)
O7	0.0298 (13)	0.252 (6)	0.0303 (13)	0.005 (3)	0.0133 (12)	0.005 (3)

### Geometric parameters (Å, °)

**Table:**

O1—C2	1.356 (4)	C9—H9C	0.9599
O1—H1	0.8200	C10—C11	1.526 (5)
O2—C4	1.349 (4)	C10—H10A	0.9700
O2—H2	0.8200	C10—H10B	0.9700
O3—C7	1.220 (4)	C11—C12	1.532 (5)
O4—C7	1.327 (4)	C11—H11A	0.9700
O4—C8	1.474 (3)	C11—H11B	0.9700
O5—C13	1.429 (4)	C12—C13	1.518 (5)
O5—H5	0.8200	C12—H12A	0.9700
O6—C14	1.443 (4)	C12—H12B	0.9700
O6—H6	0.8200	C13—C14	1.527 (4)
C1—C6	1.424 (4)	C13—H13	0.9800
C1—C2	1.426 (4)	C14—C15	1.528 (5)
C1—C7	1.470 (4)	C14—H14	0.9800
C2—C3	1.377 (4)	C15—C16	1.526 (5)
C3—C4	1.384 (4)	C15—H15A	0.9700
C3—H3	0.9300	C15—H15B	0.9700
C4—C5	1.400 (4)	C16—C17	1.507 (4)
C5—C6	1.383 (4)	C16—H16A	0.9700
C5—H5A	0.9300	C16—H16B	0.9700
C6—C18	1.497 (4)	C17—C18	1.321 (5)
C8—C9	1.492 (6)	C17—H17	0.9300
C8—C10	1.514 (5)	C18—H18	0.9300
C8—H8	0.9800	O7—H7X	0.857 (18)
C9—H9A	0.9599	O7—H7Y	0.844 (18)
C9—H9B	0.9599		
			
C2—O1—H1	109.5	C10—C11—C12	110.7 (3)
C4—O2—H2	109.5	C10—C11—H11A	109.5
C7—O4—C8	118.3 (2)	C12—C11—H11A	109.5
C13—O5—H5	109.5	C10—C11—H11B	109.5
C14—O6—H6	109.5	C12—C11—H11B	109.5
C6—C1—C2	118.0 (3)	H11A—C11—H11B	108.1
C6—C1—C7	125.7 (3)	C13—C12—C11	114.7 (3)
C2—C1—C7	116.3 (3)	C13—C12—H12A	108.6
O1—C2—C3	116.2 (2)	C11—C12—H12A	108.6
O1—C2—C1	122.4 (3)	C13—C12—H12B	108.6
C3—C2—C1	121.4 (3)	C11—C12—H12B	108.6
C2—C3—C4	119.8 (3)	H12A—C12—H12B	107.6
C2—C3—H3	120.1	O5—C13—C12	110.6 (3)
C4—C3—H3	120.1	O5—C13—C14	108.5 (2)
O2—C4—C3	121.9 (3)	C12—C13—C14	115.3 (3)
O2—C4—C5	117.9 (3)	O5—C13—H13	107.4
C3—C4—C5	120.1 (3)	C12—C13—H13	107.4
C6—C5—C4	121.3 (3)	C14—C13—H13	107.4
C6—C5—H5A	119.3	O6—C14—C13	106.2 (2)
C4—C5—H5A	119.3	O6—C14—C15	109.1 (3)
C5—C6—C1	119.3 (3)	C13—C14—C15	117.9 (3)
C5—C6—C18	117.1 (3)	O6—C14—H14	107.8
C1—C6—C18	123.2 (3)	C13—C14—H14	107.8
O3—C7—O4	122.2 (3)	C15—C14—H14	107.8
O3—C7—C1	124.0 (3)	C16—C15—C14	118.3 (3)
O4—C7—C1	113.9 (2)	C16—C15—H15A	107.7
O4—C8—C9	110.0 (3)	C14—C15—H15A	107.7
O4—C8—C10	103.8 (2)	C16—C15—H15B	107.7
C9—C8—C10	116.0 (3)	C14—C15—H15B	107.7
O4—C8—H8	108.9	H15A—C15—H15B	107.1
C9—C8—H8	108.9	C17—C16—C15	116.3 (3)
C10—C8—H8	108.9	C17—C16—H16A	108.2
C8—C9—H9A	109.5	C15—C16—H16A	108.2
C8—C9—H9B	109.5	C17—C16—H16B	108.2
H9A—C9—H9B	109.5	C15—C16—H16B	108.2
C8—C9—H9C	109.5	H16A—C16—H16B	107.4
H9A—C9—H9C	109.5	C18—C17—C16	124.6 (3)
H9B—C9—H9C	109.5	C18—C17—H17	117.7
C8—C10—C11	114.6 (3)	C16—C17—H17	117.7
C8—C10—H10A	108.6	C17—C18—C6	124.7 (3)
C11—C10—H10A	108.6	C17—C18—H18	117.6
C8—C10—H10B	108.6	C6—C18—H18	117.6
C11—C10—H10B	108.6	H7X—O7—H7Y	121 (3)
H10A—C10—H10B	107.6		
			
C6—C1—C2—O1	−178.2 (3)	C2—C1—C7—O4	161.9 (3)
C7—C1—C2—O1	2.4 (5)	C7—O4—C8—C9	84.2 (4)
C6—C1—C2—C3	2.7 (5)	C7—O4—C8—C10	−151.0 (3)
C7—C1—C2—C3	−176.7 (3)	O4—C8—C10—C11	−61.1 (4)
O1—C2—C3—C4	177.3 (3)	C9—C8—C10—C11	59.7 (4)
C1—C2—C3—C4	−3.6 (5)	C8—C10—C11—C12	157.2 (3)
C2—C3—C4—O2	−175.3 (3)	C10—C11—C12—C13	−179.1 (3)
C2—C3—C4—C5	2.6 (5)	C11—C12—C13—O5	−173.0 (3)
O2—C4—C5—C6	177.2 (3)	C11—C12—C13—C14	63.5 (4)
C3—C4—C5—C6	−0.8 (6)	O5—C13—C14—O6	74.9 (4)
C4—C5—C6—C1	−0.1 (6)	C12—C13—C14—O6	−160.5 (3)
C4—C5—C6—C18	−173.1 (3)	O5—C13—C14—C15	−47.6 (4)
C2—C1—C6—C5	−0.9 (5)	C12—C13—C14—C15	77.0 (4)
C7—C1—C6—C5	178.5 (3)	O6—C14—C15—C16	162.4 (3)
C2—C1—C6—C18	171.7 (3)	C13—C14—C15—C16	−76.5 (4)
C7—C1—C6—C18	−8.9 (5)	C14—C15—C16—C17	−63.5 (4)
C8—O4—C7—O3	−2.6 (6)	C15—C16—C17—C18	133.7 (4)
C8—O4—C7—C1	178.6 (3)	C16—C17—C18—C6	167.4 (3)
C6—C1—C7—O3	163.7 (4)	C5—C6—C18—C17	−37.0 (5)
C2—C1—C7—O3	−16.9 (5)	C1—C6—C18—C17	150.3 (4)
C6—C1—C7—O4	−17.5 (5)		

### Hydrogen-bond geometry (Å, °)

**Table:**

*D*—H···*A*	*D*—H	H···*A*	*D*···*A*	*D*—H···*A*
O1—H1···O3	0.82	1.85	2.560 (4)	144.
O2—H2···O7	0.82	1.79	2.608 (4)	172.
C3—H3···O7	0.93	2.77	3.398 (4)	125.
C9—H9B···O3	0.96	2.73	3.198 (5)	111.
O1—H1···O3^i^	0.82	2.46	3.003 (4)	125.
O7—H7Y···O2^ii^	0.84 (2)	2.01 (3)	2.798 (4)	156 (6)
O5—H5···O6^iii^	0.82	2.02	2.821 (4)	166.
O6—H6···O1^iv^	0.82	2.13	2.871 (4)	149.
C3—H3···O6^v^	0.93	2.88	3.398 (4)	116.
C10—H10B···O2^v^	0.97	2.52	3.346 (6)	144.
O7—H7X···O5^vi^	0.86 (2)	1.82 (2)	2.674 (4)	176 (4)

Symmetry codes: (i) −*x*+2, *y*, −*z*+1; (ii) −*x*+1, *y*, −*z*; (iii) −*x*+3/2, *y*−1/2, −*z*+2; (iv) −*x*+3/2, *y*+1/2, −*z*+1; (v) −*x*+3/2, *y*−1/2, −*z*+1; (vi) *x*, *y*, *z*−1.
